# Nutritional and hematological determinants of immune reconstitution and development of a predictive model among virologically suppressed people living with HIV

**DOI:** 10.3389/fnut.2026.1936337

**Published:** 2026-09-28

**Authors:** Ke Xin, Lijing Wang, Yuxin Feng, Jianhua Lu, Xiaojing Qu, Yingxia Li, Xingyi Yao, Beilei Li, Erhei Dai, Yuling Wang, Huixia Gao

**Affiliations:** 1 Medical Technology College, Hebei Medical University, Shijiazhuang, China; 2 The Fifth Hospital of Shijiazhuang City, Shijiazhuang, Hebei Province, China

**Keywords:** antiretroviral therapy, Cox regression, HIV, immunological non-response, predictive model

## Abstract

**Background:**

Despite effective viral suppression achieved through antiretroviral therapy (ART), a substantial proportion of people living with HIV (PLWH) fail to achieve adequate immune reconstitution and remain at increased risk of adverse clinical outcomes.

**Methods:**

A retrospective cohort study was conducted among PLWH in Hebei Province, China, who received long-term ART and achieved sustained virological suppression. Immune reconstitution was defined as achieving a CD4 + T cell count ≥350 cells/μL during follow-up. Kaplan–Meier analysis and Cox proportional hazards regression were performed to evaluate the dynamics of immune recovery and identify independent predictors associated with time to immune reconstitution. A prognostic model was developed and evaluated using Harrell’s concordance index (C-index), time-dependent receiver operating characteristic (ROC) curves, calibration analysis, and 1,000-times bootstrap internal validation.

**Results:**

During a median follow-up of 4.02 years (95% CI: 4.00–4.04 years), 1,651 (76.9%) participants achieved immune reconstitution, whereas 495 (23.1%) did not achieve immune reconstitution. The probability of immune recovery increased rapidly during the first year after ART initiation and subsequently plateaued. Multivariable Cox regression analysis showed that older age, advanced WHO clinical stage, and lower baseline CD4 + T cell count were independently associated with a reduced probability of immune reconstitution. In addition, overweight status (BMI ≥ 24 kg/m2; HR = 1.12, 95% CI: 1.02–1.23, *p* = 0.021) and higher baseline hemoglobin levels (HR = 1.004, 95% CI: 1.001–1.007, *p* = 0.016) were independently associated with improved immune recovery after adjustment for established clinical and immunological factors. The prediction model demonstrated good discrimination (C-index = 0.713), with time-dependent AUCs of 0.849, 0.874, and 0.850 at 1, 2, and 4 years, respectively. Calibration analysis showed good agreement between predicted and observed immune recovery probabilities.

**Conclusion:**

Approximately one-quarter of virologically suppressed PLWH did not achieve immune reconstitution after long-term ART. Immune recovery occurred predominantly during the first year after ART initiation. In addition to established predictors such as age, baseline CD4 + T cell count, and WHO clinical stage, BMI and hemoglobin levels may serve as readily obtainable clinical indicators reflecting nutritional reserve and overall health status. The developed prediction model may provide a practical approach for identifying individuals with delayed immune recovery and supporting individualized clinical monitoring.

## Introduction

The widespread implementation of antiretroviral therapy (ART) has substantially improved the prognosis of people living with HIV (PLWH). However, despite achieving sustained virological suppression, a considerable proportion of individuals fail to achieve adequate immune restoration, a condition commonly referred to as immunological non-response (INR). Persistent incomplete immune recovery has been associated with increased risks of opportunistic infections, non-AIDS-defining diseases, and mortality, emphasizing the clinical importance of identifying individuals at risk of poor immune recovery despite effective viral control (1).

Previous studies have estimated that approximately 15–30% of PLWH experience incomplete immune reconstitution after ART initiation. The mechanisms underlying suboptimal immune recovery are complex and involve multiple host, treatment, and immunological factors. Older age, delayed ART initiation, low baseline CD4^+^ T cell count, impaired hematopoietic function, reduced thymic activity, persistent immune activation, inflammatory responses, lymphoid tissue fibrosis, and metabolic disturbances have all been implicated in impaired immune restoration. However, among individuals who achieve long-term virological suppression, the contribution of readily available clinical characteristics to long-term immune recovery remains incompletely understood (2).

In addition to traditional immunological and virological determinants, nutritional and hematological status has increasingly been recognized as an important contributor to long-term outcomes among PLWH receiving ART. Body mass index (BMI), as a simple and routinely available indicator of nutritional reserve, reflects overall energy balance and metabolic status, which may influence immune restoration through effects on inflammation, immune homeostasis, and lymphocyte function. Both undernutrition and metabolic abnormalities have been associated with persistent systemic inflammation, impaired immune cell function, and suboptimal CD4 + T-cell recovery (3). Hematological parameters, particularly hemoglobin (HGB) levels, may also provide important information regarding the host environment underlying immune recovery. Hemoglobin reflects not only hematopoietic function and oxygen-carrying capacity but also nutritional status, chronic inflammation, and overall physiological condition. Anemia is frequently observed among PLWH and has been associated with immune activation, disease progression, and mortality, suggesting that hematological status may influence the capacity for immune restoration after ART initiation (4). Despite these observations, previous prediction models of immune recovery have predominantly focused on baseline immunological and virological characteristics, particularly CD4 + T-cell-related parameters, while the contribution of routinely available nutritional and hematological indicators has been insufficiently explored. Therefore, integrating these accessible clinical characteristics into predictive models may provide additional value for identifying individuals at risk of incomplete immune recovery (5).

Several predictive models have been developed to evaluate immune recovery among PLWH receiving ART. However, most existing studies have focused on early immune responses after ART initiation, CD4^+^ T cell slope changes, or short-term immune recovery outcomes (6, 7). Given that immune recovery follows a nonlinear trajectory characterized by rapid CD4^+^ T cell increases during the early phase of ART followed by gradual slowing and stabilization after several years, prediction tools specifically designed for individuals with sustained virological suppression and long-term ART exposure remain limited. Furthermore, few studies have integrated longitudinal immune recovery patterns with nutritional and hematological characteristics to develop a clinically applicable prediction model (8, 9).

Therefore, this study aimed to characterize long-term immune reconstitution patterns among PLWH with sustained virological suppression after ART and to develop a clinically applicable prediction model incorporating routinely available clinical, nutritional, and hematological characteristics to identify individuals at increased risk of incomplete immune recovery.

## Methods

### Participants and study design

This retrospective cohort study included HIV-infected individuals who received treatment in Hebei Province, China from March 2013 to December 2021. Participants were followed until December 2025. A total of 3,323 patients were initially screened for eligibility.

### Definition of key outcome

Regarding the CD4^+^ T cell design of this study, WHO and international guidelines recognize CD4^+^ T cell thresholds as clinically relevant indicators of immune status, while definitions of immune recovery vary across studies, no universally accepted criterion has been established. In the context of long-term ART with sustained virological suppression, a CD4^+^ T cell threshold of 350 cells/μL represents a clinically meaningful level of immune restoration, reflecting recovery beyond severe immunodeficiency and reduced risk of HIV-related complications. This threshold is also consistent with the Chinese expert consensus on immune reconstitution in HIV-infected individuals. Therefore, immune reconstitution was defined as achieving a CD4^+^ T cell≥350 cells/μL during follow-up in this study.

Specifically, INR was defined as follows: patients receiving standardized ART for ≥4 years, achieving sustained virological suppression (viral load [VL] < 50 copies/mL) for ≥3 years without virological rebound, and maintaining a peripheral blood CD4^+^ T cell count <350 cells/μL. In addition, other known causes of persistent lymphocytopenia were excluded, including primary immunodeficiency disorders, chronic viral infections, hematologic malignancies, and long-term immunosuppressive therapy (10).

Inclusion Criteria: 1. Confirmed HIV antibody positivity by both screening and confirmatory testing; 2. Continuous ART for ≥4 years; 3. Sustained virological suppression (VL < 50 copies/mL) for ≥3 consecutive years without virological rebound during treatment.

Exclusion Criteria: 1. Baseline CD4^+^ T cell count >500 cells/μL before ART initiation, (as these individuals had already achieved favorable immune status at baseline and were unsuitable for evaluating immune reconstitution dynamics); 2. Loss to follow-up, treatment interruption, or virological rebound during treatment; 3. Missing key baseline information, including baseline CD4^+^ T cell count, baseline viral load, and initial ART regimen.

### Nutritional and hematological assessment

Baseline nutritional and hematological parameters were collected before ART initiation or at the first clinical evaluation before treatment. Nutritional indicators included body mass index (BMI), serum albumin, total cholesterol, triglycerides, and other routinely measured biochemical parameters reflecting nutritional status. BMI was calculated as weight (kg) divided by height squared (m^2^) and categorized according to Chinese adult criteria.

Hematological indicators included hemoglobin (HGB), platelet count, white blood cell count, lymphocyte count, and related hematological parameters. These indicators were selected because nutritional status and hematological abnormalities have been reported to influence immune recovery among people living with HIV receiving ART. In addition, these parameters are routinely available in clinical practice, making them clinically applicable for risk stratification and prediction modeling.

### Statistical analysis

Baseline demographic and clinical characteristics were summarized using descriptive statistics. Categorical variables were presented as frequencies and percentages. Between-group comparisons were performed using the chi-square test or Fisher’s exact test for categorical variables and appropriate parametric or non-parametric tests for continuous variables. Standardized mean differences (SMDs) were calculated as supplementary measures of baseline differences between groups.

Missing data proportions were calculated for all candidate variables before model development. Variables with missing key baseline information, including baseline CD4^+^ T cell count, baseline viral load, and initial ART regimen, were excluded because these parameters were essential for eligibility assessment and model construction. Given the nature of these exclusions, complete-case analysis was performed without multiple imputation.

Candidate predictors were selected *a priori* based on previous literature, clinical relevance, biological plausibility, and routine availability in clinical practice. Univariable Cox proportional hazards regression analyses were performed to evaluate associations between candidate predictors and immune reconstitution. Variables with *p* < 0.10 in univariable analyses were considered for inclusion in the multivariable model, while clinically established predictors were retained regardless of statistical significance. The number of outcome events per variable (EPV) was calculated to evaluate the adequacy of the sample size for multivariable Cox regression modeling.

Kaplan–Meier survival analysis was used to estimate the cumulative probability of immune reconstitution. Differences between groups were assessed using the log-rank test. Participants who did not achieve immune reconstitution during the observation period (*n* = 495) were treated as right-censored observations at the end of follow-up, because the event had not occurred by the last available assessment.

Cox proportional hazards regression models were used to identify factors associated with immune reconstitution, and hazard ratios (HRs) with corresponding 95% confidence intervals (CIs) were calculated. The proportional hazards assumption was evaluated using Schoenfeld residual tests. Variables violating the proportional hazards assumption but considered clinically important were retained, and their HRs were interpreted as average effects throughout the follow-up period.

Model performance was evaluated using Harrell’s C-index, time-dependent receiver operating characteristic (ROC) curves, calibration plots, calibration slope, and Brier scores. The confidence intervals of time-dependent area under the curve (AUC) values were estimated using bootstrap resampling. Internal validation was performed using 1,000 bootstrap resamples to correct optimism and evaluate model stability. Based on the independent predictors identified from the multivariable Cox regression analysis, a prognostic nomogram was developed to provide individualized estimates of the probability of immune reconstitution at 1, 2, and 4 years after ART initiation. Decision curve analysis was conducted to assess the clinical utility of the prediction model by calculating net benefit across different threshold probabilities at 1, 2, and 4 year prediction horizons.

Sensitivity analysis was performed using a more stringent immune recovery threshold (CD4^+^ T cell count ≥500 cells/μL) to evaluate the robustness of the findings. All statistical analyses were performed using SPSS version 27.0 and R version 4.5.2. All statistical tests were two-sided, and *p* < 0.05 was considered statistically significant.

## Results

### Patient selection and baseline characteristics

Between March 2013 and December 2021, a total of 3,343 people living with HIV (PLWH) receiving ART were retrospectively screened for eligibility. Among them, 1,197 individuals were excluded due to insufficient treatment duration, incomplete viral load data, or missing key baseline information.

Ultimately, 2,146 HIV-infected patients met the inclusion criteria and were enrolled in the study (Figure 1). Of these participants, 495 patients (23.1%) were classified into the INR group, whereas 1,651 patients (76.9%) achieved IR group. The study population was predominantly male (91.7%), with most participants aged 31–50 years (51.4%) or ≥50 years (37.9%). Homosexual transmission was the primary route of HIV acquisition, accounting for 73.0% of cases. Baseline immunological status indicated substantial immune suppression in the cohort. Specifically, 32.4% of patients had baseline CD4^+^ T cell counts <200 cells/μL, and 61.2% had a CD4/CD8 ratio <0.3. Regarding treatment regimens, non-nucleoside reverse transcriptase inhibitor (NNRTI)-based therapy was the predominant ART strategy, accounting for 87.5% of cases.

**Figure 1:**
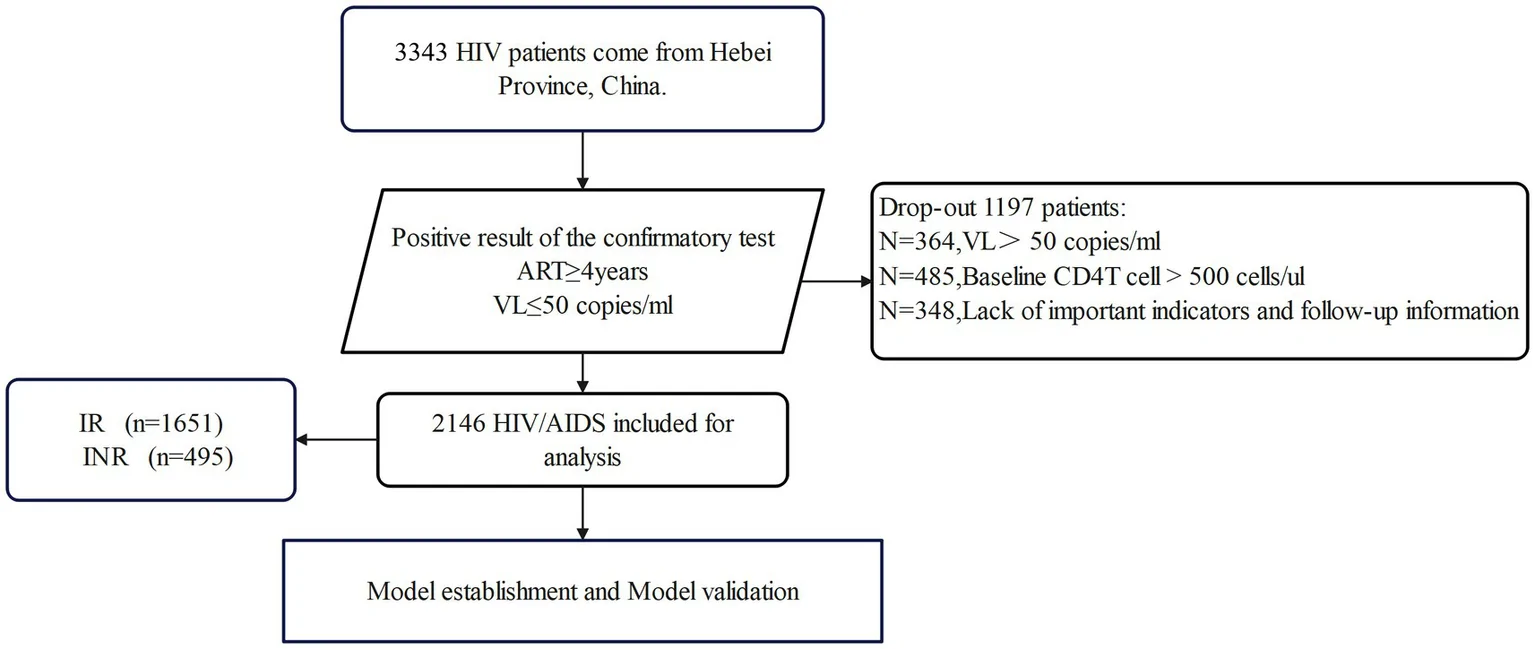
Flowchart of study selection for the present study. This flowchart provides a detailed description of the inclusion and exclusion criteria for the cohort: the initially screened patients (*n* = 3,343), those excluded for various reasons (*n* = 1,197), and the final cohort that was included (*n* = 2,146).

Comparative analyses between the two groups demonstrated that patients in the INR group were significantly older than those in the IR group, with a higher proportion aged ≥50 years (51.1% vs. 33.9%, *p* < 0.001). The INR group also exhibited a markedly higher prevalence of advanced WHO clinical stage III/IV disease (66.1% vs. 18.4%, *p <* 0.001) and poorer baseline immune status, as reflected by a greater proportion of patients with baseline CD4^+^ T cell counts <200 cells/μL (71.3% vs. 21.7%, *p <* 0.001) and CD4/CD8 ratios <0.3 (82.8% vs. 54.8%, *p <* 0.001). In addition, significant differences in BMI distribution were observed between the two groups (*p <* 0.001). In the INR group, a higher proportion of patients had normal body weight (BMI 18.5–23.9 kg/m^2^) compared with the IR group (63.8% vs. 56.5%). Conversely, overweight (BMI 24–27.9 kg/m^2^) and obese (BMI ≥ 28 kg/m^2^) individuals were more prevalent in the IR group than in the INR group (8.5% vs. 4.8 and 26.9% vs. 19.2%, respectively). Significant between-group differences were also observed with respect to marital status and timely initiation of treatment (both *p <* 0.001). However, no statistically significant differences were identified regarding sex, route of HIV transmission, baseline viral load, or initial ART regimen (all *p* > 0.05) (Table 1).

**Table 1:** Demographic and clinical characteristics of patients with IR and INR.

Characteristic	Overall (*n* = 2,146)	INR (*n* = 495)	IR (*n* = 1,651)	*p* value	SMD
Age				<0.001	0.378
≤30	229 (10.7%)	29 (5.9%)	200 (12.1%)		
31–50	1,104 (51.4%)	213 (43.0%)	891 (54.0%)		
>50	813 (37.9%)	253 (51.1%)	560 (33.9%)		
Sex				0.064	0.102
Male	1967 (91.7%)	464 (93.7%)	1,503 (91.0%)		
Female	179 (8.3%)	31 (6.3%)	148 (9.0%)		
BMI (kg/m^2^)				<0.001	0.213
<18.5	184 (8.6%)	51 (10.3%)	133 (8.1%)		
18.5–23.9	1,258 (58.6%)	325 (65.7%)	933 (56.5%)		
24–27.9	164 (7.6%)	24 (4.8%)	140 (8.5%)		
≥28	540 (25.2%)	95 (19.2%)	445 (26.9%)		
Marital status				<0.001	0.344
Unmarried	824 (38.4%)	129 (26.1%)	695 (42.1%)		
Married	1,087 (50.7%)	304 (61.4%)	783 (47.4%)		
Divorced/Widowed	235 (10.9%)	62 (12.5%)	173 (10.5%)		
Infection route				0.4	0.064
Heterosexual	506 (23.6%)	124 (25.1%)	382 (23.1%)		
Homosexual	1,566 (73.0%)	351 (70.9%)	1,215 (73.6%)		
Others/Unclear^a^	74 (3.4%)	20 (4.0%)	54 (3.3%)		
Treatment delay				<0.001	0.201
No	623 (29.0%)	110 (22.2%)	513 (31.1%)		
Yes	1,523 (71.0%)	385 (77.8%)	1,138 (68.9%)		
WHO clinical stage				<0.001	0.981
Stage I/II	1,515 (70.6%)	168 (33.9%)	1,347 (81.6%)		
Stage III/IV	631 (29.4%)	327 (66.1%)	304 (18.4%)		
CD4^+^ T cell (cells/μL)				<0.001	1.012
<200	695 (32.4%)	353 (71.3%)	342 (20.7%)		
200–350	845 (39.4%)	116 (23.4%)	729 (44.2%)		
351–500	606 (28.2%)	26 (5.3%)	580 (35.1%)		
CD4/CD8				<0.001	0.643
<0.3	1,314 (61.2%)	410 (82.8%)	904 (54.8%)		
0.3–0.5	589 (27.4%)	67 (13.5%)	522 (31.6%)		
>0.5	243 (11.3%)	18 (3.7%)	225 (13.6%)		
Initial ART regimen				0.064	0.100
NRTI+INSTI	206 (9.6%)	59 (11.9%)	147 (8.9%)		
NRTI+NNRTI	1878 (87.5%)	421 (85.1%)	1,457 (88.2%)		
NRTI+PI	62 (2.9%)	15 (3.0%)	47 (2.8%)		

NNRTIs, nonnucleoside reverse transcriptase inhibitors; PIs, protease inhibitors; INSTI, integrase strand transfer inhibitors; Treatment delay, the interval between the date of confirming HIV positivity and the date of starting antiviral treatment is less than 30 days.

a Others/Unclear included blood transfusion, mother-to-child transmission, and unknown.

### Trajectory of immune reconstitution and stratified survival analysis

Kaplan–Meier analysis was performed to estimate the cumulative probability and time distribution of immune reconstitution after ART initiation (Figure 2). Among the 2,146 participants included in the analysis, 1,651 achieved immune reconstitution during follow-up. The total observation time was 3,511.8 person-years, corresponding to an incidence rate of immune reconstitution of 46.5 per 100 person-years (95% CI: 44.3–48.8).

**Figure 2:**
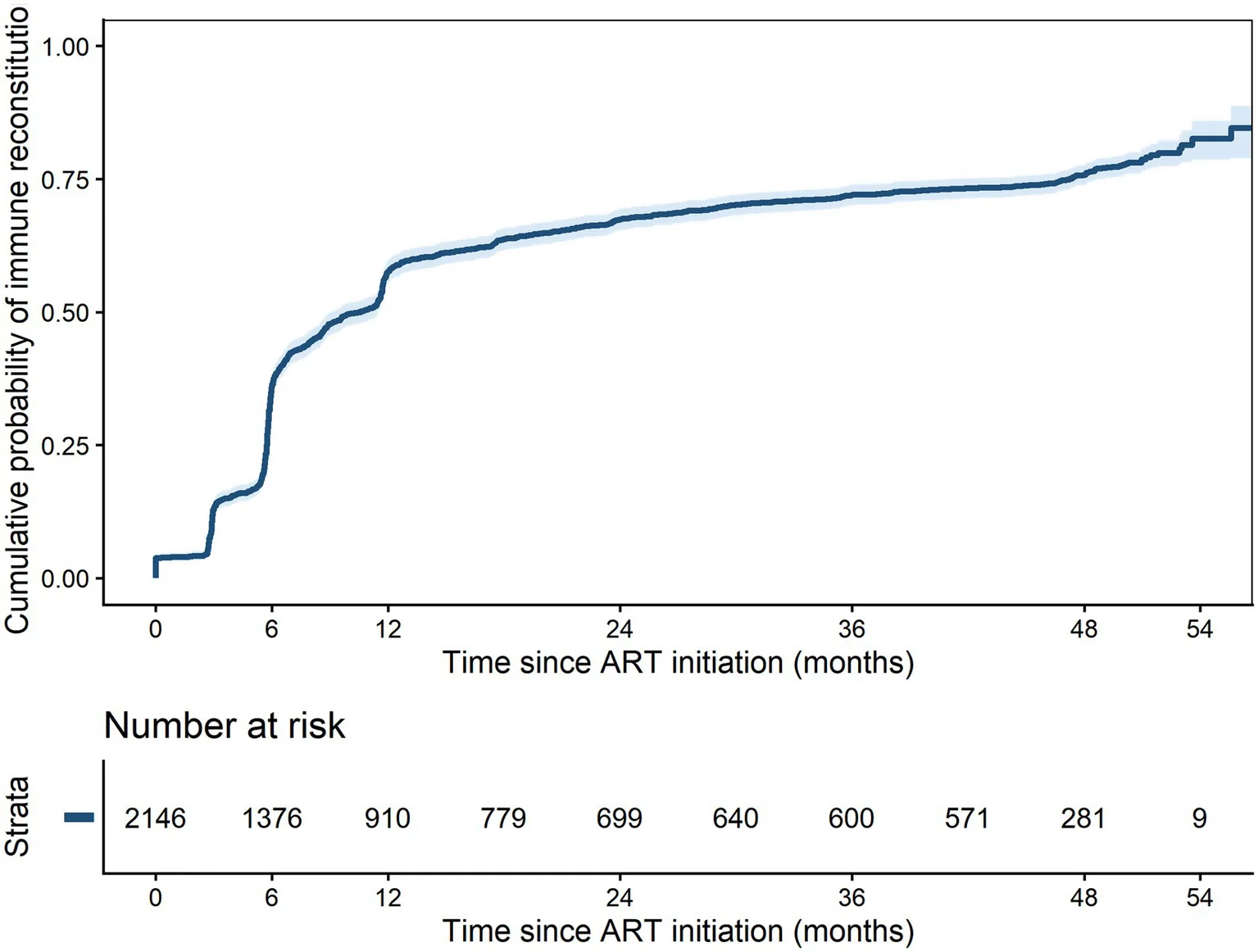
The Kaplan–Meier curve shows the cumulative probability of immune reconstitution in the overall study population after the initiation of ART. The x-axis represents time since ART initiation (months), and the y-axis represents the estimated cumulative probability of immune reconstitution. The solid line represents the Kaplan–Meier estimate, with the shaded area indicating the 95% confidence interval. The median time to immune reconstitution was 10.5 months (95% CI: 9.1–11.4). The number-at-risk table displays the number of participants who remained under observation and had not yet achieved immune reconstitution at each time point.

The cumulative probability of immune reconstitution increased rapidly during the early period after ART initiation. The estimated probability of immune recovery reached 38.0% at 6 months, increased to 62.0% at 12 months, and approximately 70.0% at 24 months. Subsequently, the slope of the curve became markedly attenuated, indicating a plateau phase of immune recovery.

The median time to immune reconstitution was 10.5 months (95% CI: 9.1–11.4). The majority of immune recovery events occurred within the first year after ART initiation, suggesting that the early ART period represents a critical window for immune restoration.

Kaplan–Meier analyses were further performed according to baseline CD4^+^ T cell count, WHO clinical stage, BMI category, and hemoglobin level. Significant differences in immune recovery trajectories were observed across these stratified groups (log-rank test, all *p* < 0.001; Figure 3).

**Figure 3:**
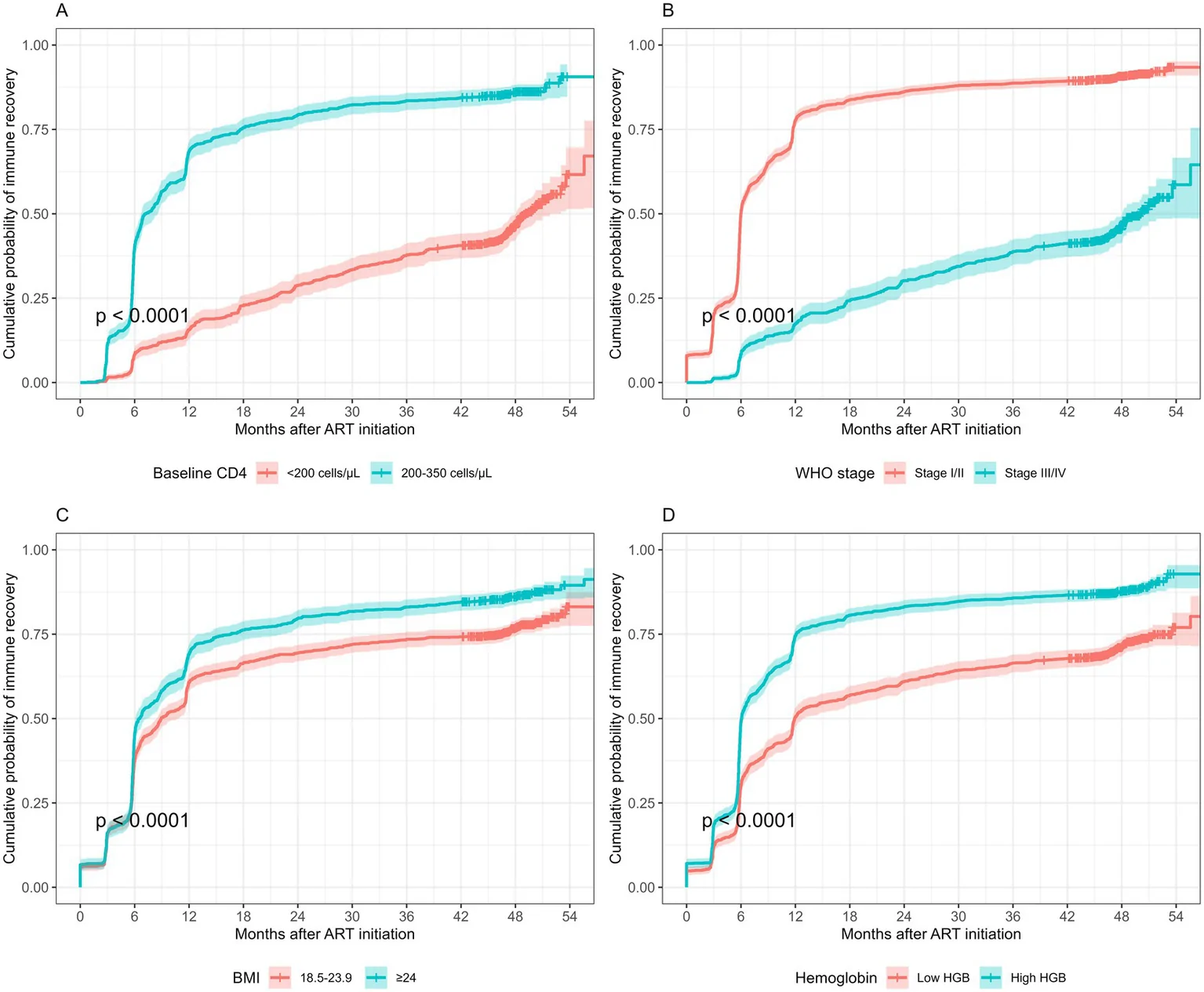
K-M survival curve subgroup plot. The Kaplan–Meier curve depicting the cumulative probability of immune reconstitution after the initiation of antiretroviral therapy (ART) based on baseline CD4 + T-cell count **(A)**, WHO clinical stage **(B)**, BMI **(C)**, and HGB **(D)**.

Patients with higher baseline CD4^+^ T cell counts demonstrated a significantly greater probability of immune recovery compared with those with lower baseline CD4^+^ T cell counts. Similarly, individuals with WHO clinical stage I/II showed faster and more sustained immune recovery than those with stage III/IV disease. Patients with BMI ≥ 24 kg/m^2^ and higher baseline hemoglobin levels showed higher cumulative probabilities of immune recovery. However, these associations should be interpreted as indicators of nutritional and hematological status rather than direct therapeutic effects.

### Cox regression analysis of factors associated with immune reconstitution following antiretroviral therapy

Univariable Cox proportional hazards regression analysis was performed to identify factors associated with immune reconstitution. Age at ART initiation, BMI category, transmission route, marital status, WHO clinical stage, initial ART regimen, timing of ART initiation, baseline CD4/CD8 ratio, baseline CD4^+^ T cell count, baseline viral load, hemoglobin level, platelet count, aspartate aminotransferase (AST), and total bilirubin (T.BIL) were significantly associated with immune recovery in univariable analysis (all *p* < 0.05; Table 2). Variables with *p* < 0.10 in univariable analysis, together with clinically established predictors, were considered for multivariable Cox regression analysis.

**Table 2:** Results of univariate Cox regression analysis.

Variable	Ref.	HR (95% CI)	*P* value
Age 31–50	Age ≤30	0.82 (0.72–0.94)	<0.001
Age >50		0.56 (0.48–0.64)	<0.001
Sex Male	Female	0.9 (0.77–1.05)	0.168
BMI ≥ 24 (kg/m^2^)	BMI 18.5–23.9	1.23 (1.11–1.36)	<0.001
BMI <18.5		0.86 (0.73–1.02)	0.075
Homosexual	Heterosexual	1.12 (1.01–1.25)	0.027
Others/Unclear		0.95 (0.74–1.23)	0.718
Unmarried	Married	1.45 (1.32–1.59)	<0.001
Divorced /Widowed		1.07 (0.93–1.25)	0.339
WHO stage III/IV	I/II	0.23 (0.2–0.26)	<0.001
NRTI+INSTI	NRTI+NNRTI	0.74 (0.63–0.86)	<0.001
NRTI+PI		0.87 (0.67–1.14)	0.310
Delayed ART	Non-delayed treatment	1.17 (1.07–1.29)	<0.001
CD4/CD8<0.3	CD4/CD8>0.5	0.38 (0.34–0.43)	<0.001
CD4/CD80.3–0.5		0.81 (0.71–0.91)	<0.001
CD4^+^ T cell <200 (cells/μL)	CD4^+^ T cell 351–499	0.14 (0.12–0.16)	<0.001
CD4^+^ T cell 200–350		0.5 (0.44–0.56)	<0.001
WBC (10^9^/L)		0.99 (0.97–1.02)	0.705
HGB (g/L)		1.19 (1.16–1.22)	<0.001
PLT (10^9^/L)		1.19 (1.13–1.26)	<0.001
TC (mmol/L)		1 (0.99–1.01)	0.841
TG (mmol/L)		1.01 (0.96–1.06)	0.677
AST (U/L)		0.93 (0.9–0.97)	<0.001
T.BIL (μmol/L)		1.14 (1.1–1.19)	<0.001

WBC, white blood cell; HGB, hemoglobin; PLT, platelet; TC, total cholesterol; TG, triglyceride; AST, aspartate transaminase; T.BIL, total bilirubin.

After adjustment for potential confounding factors, older age, advanced WHO clinical stage, lower baseline CD4^+^ T cell count, overweight BMI category, and higher hemoglobin levels remained independently associated with immune reconstitution (Table 3). The effect size of hemoglobin was statistically significant but relatively modest (HR = 1.004, 95% CI:1.001–1.007, *p* = 0.016), suggesting that hemoglobin may primarily represent underlying hematological reserve rather than a direct determinant of immune recovery.

**Table 3:** Results of multivariate Cox regression analysis.

Variable	Ref.	Beta	*P* value	HR (95% CI)
Age >50	Age ≤30	−0.280	<0.001	0.76 (0.65–0.88)
BMI ≥ 24 (kg/m^2^)	BMI 18.5–23.9	0.113	0.021	1.12 (1.02–1.23)
WHO Stage III/IV	I/II	−0.423	<0.001	0.66 (0.53–0.81)
CD4^+^ T cell<200 (cells/μL)	CD4^+^ T cell 351–499	−1.489	<0.001	0.23 (0.18–0.28)
CD4^+^ T cell 200–350		−0.667	<0.001	0.51 (0.45–0.58)
CD4/CD8>0.5	CD4/CD8<0.3	0.157	0.024	1.17 (1.02–1.34)
HGB (g/L)		0.037	<0.001	1.04 (1.01–1.07)

The final model included 8 effective parameters and 1,651 immune reconstitution events, resulting in an event-per-variable (EPV) ratio of 206, supporting adequate model stability.

### Model validation and predictive performance

PH assumes that the global test *p* > 0.05; since CD4^+^ T cell count and WHO stage show non-proportional risks, the HR is interpreted as the average effect over the entire follow-up period. Collinearity assessment demonstrated that all variance inflation factors (VIFs) were <5, indicating no substantial multicollinearity among the included predictors.

The final multivariable Cox regression model showed significant overall predictive performance (likelihood ratio test: χ^2^ = 1,094, df = 21, *p* < 0.001). The model demonstrated acceptable discrimination, with a Harrell’s concordance index (C-index) of 0.713 (SE = 0.006).

Time-dependent ROC analysis further demonstrated good discriminative ability of the prediction model across different follow-up horizons. The model achieved AUC values of 0.849 (95% CI: 0.814–0.870), 0.874 (95% CI: 0.850–0.887), and 0.850 (95% CI: 0.818–0.864) at 1, 2, and 4 years after ART initiation, respectively (Figure 4).

**Figure 4:**
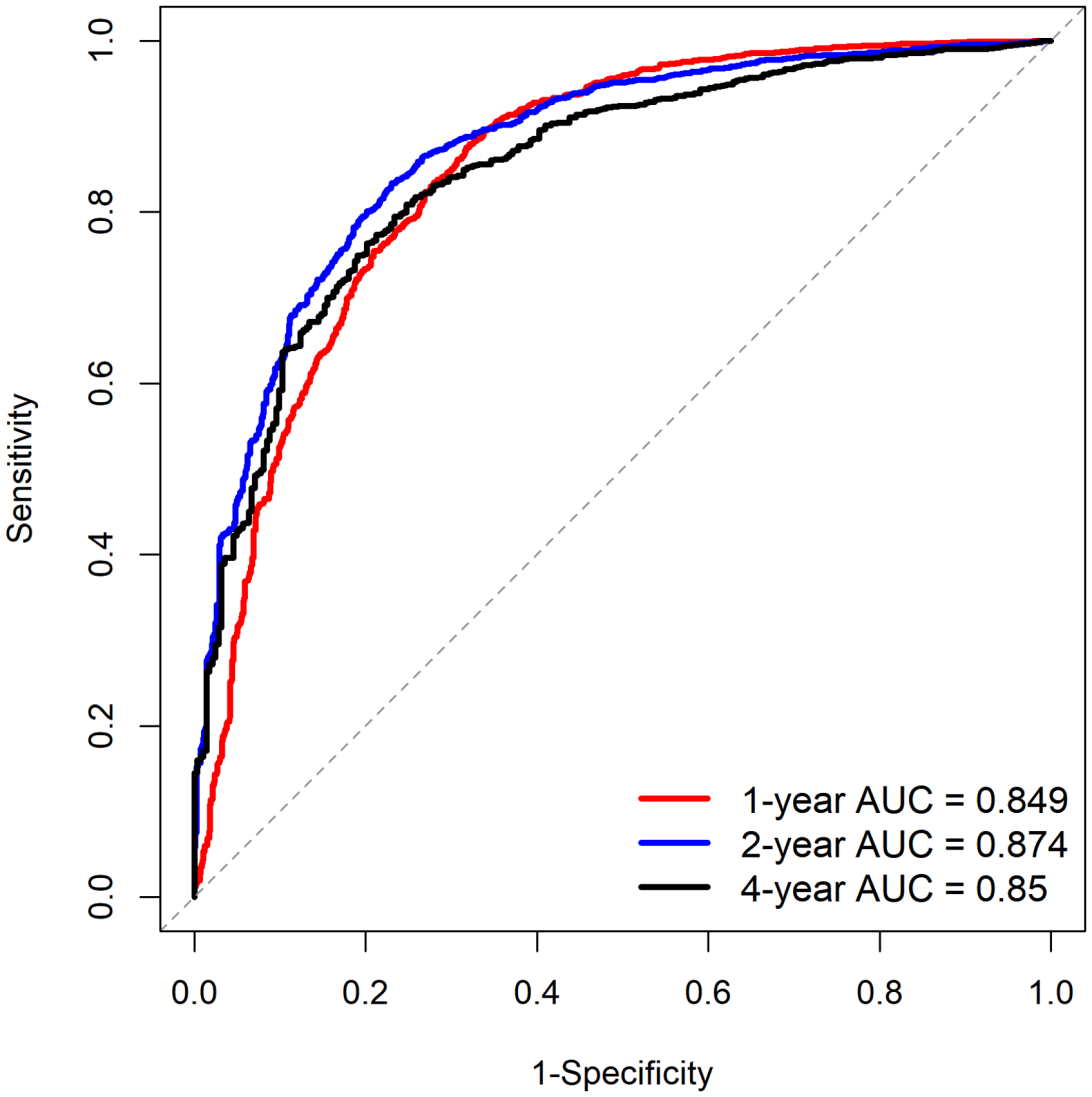
Time-dependent ROC analysis for 1, 2, and 4 years of immune reconstitution for PLWH after ART initiation. The x-axis represents the false positive rate (1-specificity), while the y-axis shows the true positive rate (sensitivity). An AUC value of 1 indicates perfect discrimination, while a value of 0.5 suggests no discriminative ability. The closer the AUC is to 1, the better the model’s performance.

The nomogram incorporated five clinically available predictors, including age, WHO clinical stage, baseline CD4^+^ T cell count, BMI, and hemoglobin level. By summing the points assigned to each predictor, clinicians can estimate an individual patient’s probability of achieving immune reconstitution at different time points after ART initiation (Figure 5).

**Figure 5:**
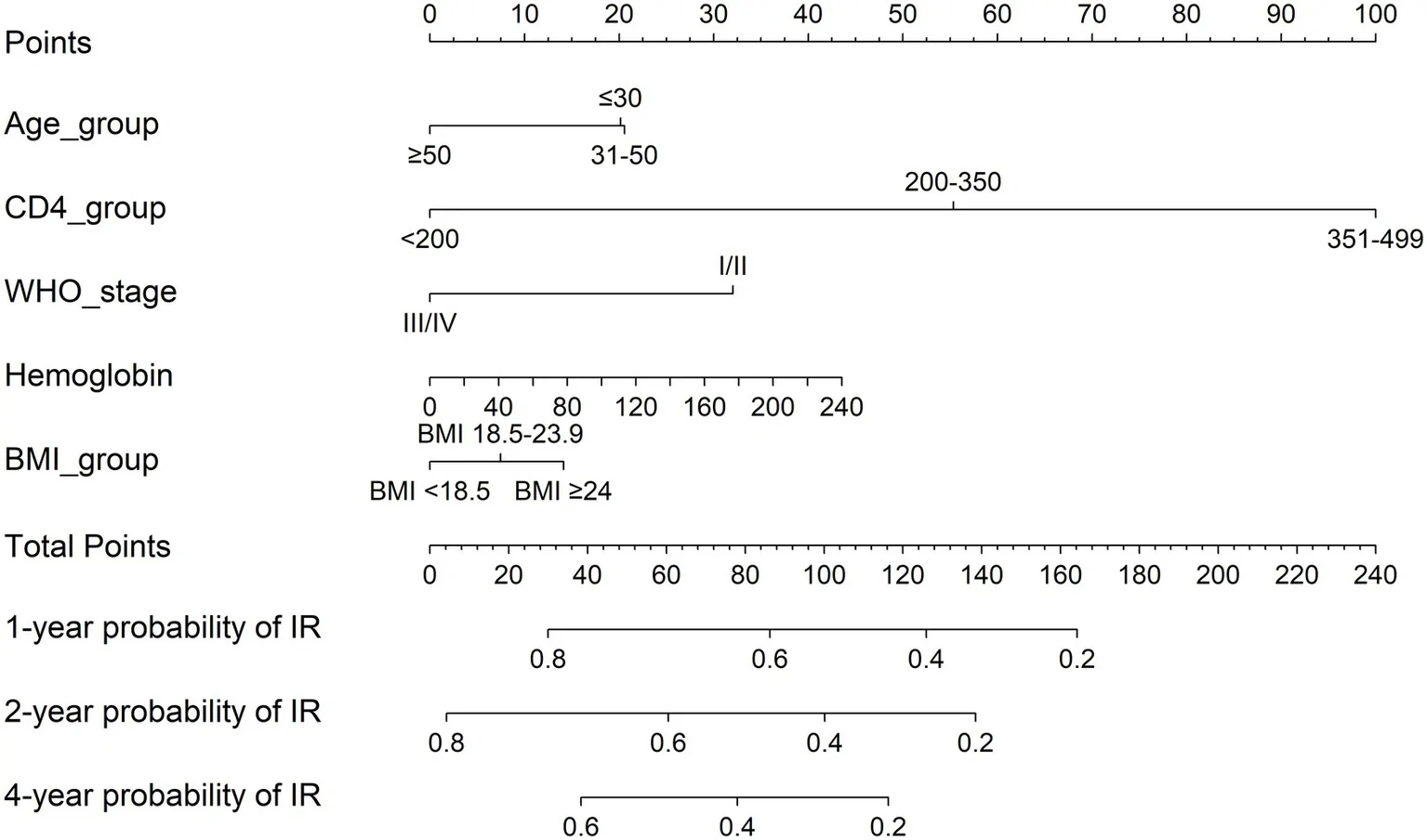
Nomogram for predicting the probability of immune reconstitution among virologically suppressed people living with HIV after antiretroviral therapy. The nomogram was developed based on the independent predictors identified from the multivariable Cox proportional hazards regression model, including age, WHO clinical stage, baseline CD4 + T-cell count, body mass index (BMI), and hemoglobin level. Each predictor was assigned a corresponding point value, and the sum of all points was used to estimate the individualized probability of achieving immune reconstitution at 1, 2, and 4 years after ART initiation. Higher total points corresponded to different predicted probabilities of immune recovery according to the time-specific survival function derived from the Cox model.

After internal verification by Bootstrap, the model demonstrated limited optimism after correction, with an optimism-corrected C-index of 0.711 (95% CI: 0.698–0.720) and a corrected Dxy discrimination index of 0.418. Calibration analysis showed good agreement between predicted and observed probabilities, with a calibration slope of 1.00 (95% CI: 0.93–1.07), suggesting no substantial overestimation or underestimation of immune reconstitution probabilities. Furthermore, the model demonstrated low prediction errors, with Brier scores of 0.036 at 1, 2, and 4 years after ART initiation.

Decision curve analysis was conducted to evaluate the potential clinical utility of the prediction model (Figure 6). Across a clinically relevant range of threshold probabilities (0–50%), the model consistently provided greater net benefit compared with the treat-all and treat-none strategies.

**Figure 6:**
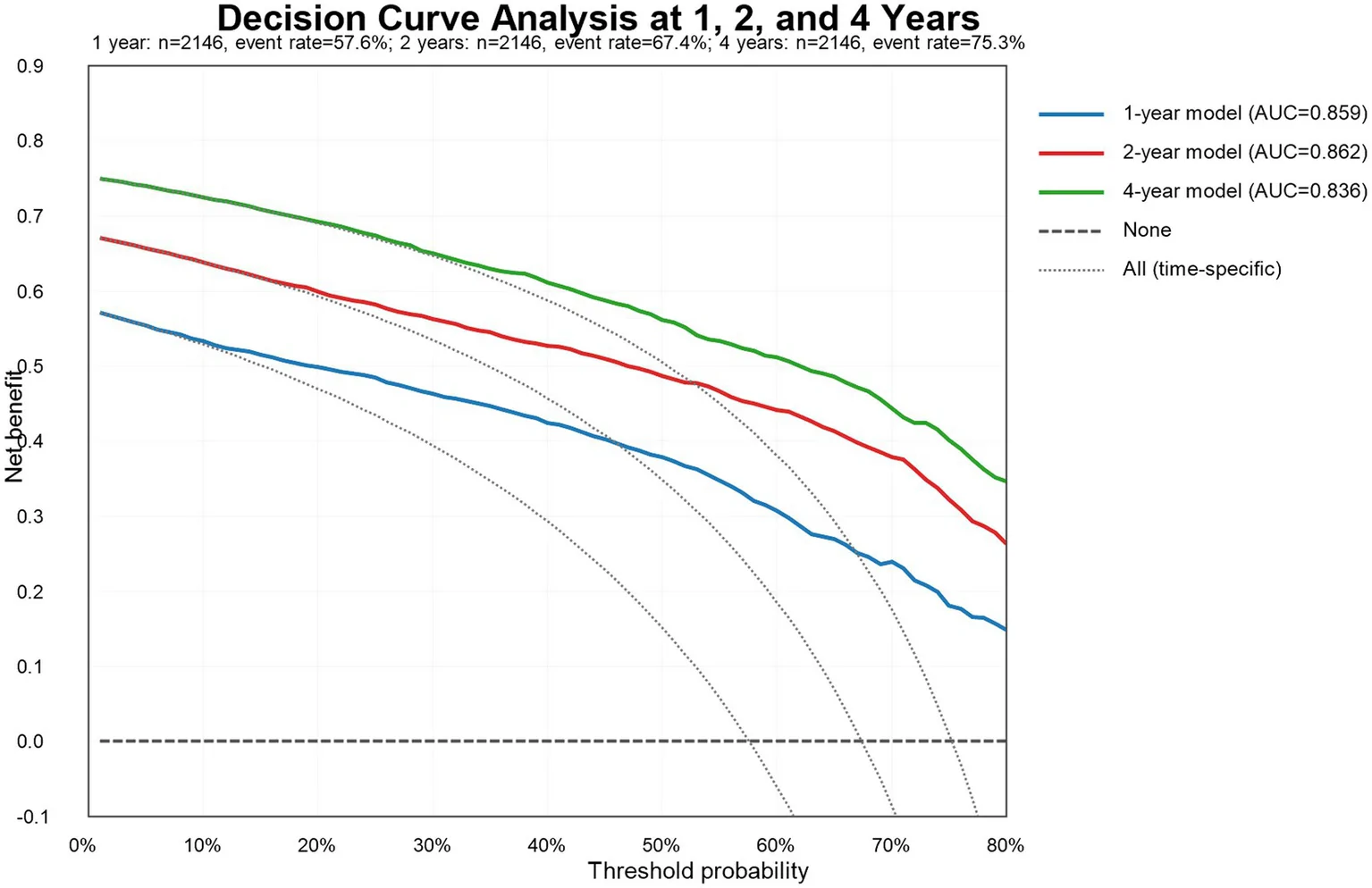
Decision curve analysis of the prediction model for immunological non-response. Decision curve analysis showed that the prediction model achieved higher net benefit than the treat-all and treat-none strategies across a clinically relevant threshold probability range (0–50%), indicating potential clinical utility for identifying PLWH at high risk of immunological non-response.

### Sensitivity analysis

The results were generally consistent with the primary analysis. Older age, advanced WHO clinical stage, and lower baseline CD4^+^ T cell count remained independently associated with delayed immune recovery. BMI ≥ 24 kg/m^2^ and higher hemoglobin levels showed similar directions of association, although effect estimates were slightly attenuated. The predictive model demonstrated comparable discrimination performance (C-index = 0.705), supporting the robustness of the primary findings.

## Discussion

Multivariable Cox regression analysis identified age, WHO clinical stage, baseline CD4^+^ T cell count, BMI, and hemoglobin level as independent factors associated with immune reconstitution. Consistent with previous studies, lower baseline CD4^+^ T cell count was strongly associated with impaired immune recovery, indicating that the degree of immunological damage before ART initiation largely determines subsequent restoration potential (10). Likewise, advanced WHO clinical stage and older age were associated with poorer immune reconstitution, which may reflect more severe disease progression, prolonged immune system injury, thymic involution, and reduced regenerative capacity (11, 12).

Notably, beyond established immunological and clinical predictors, BMI and hemoglobin level showed significant associations with immune recovery. Although baseline hemoglobin level remained independently associated with immune reconstitution after adjustment, the effect size was relatively small. Therefore, hemoglobin should be interpreted primarily as a readily available clinical marker reflecting overall physiological status rather than a direct therapeutic target for improving immune restoration (9, 10). Anemia is common among PLWH and has been associated with immune activation, opportunistic infections, and adverse clinical outcomes. Chronic inflammation, nutritional deficiencies, and impaired bone marrow function may contribute to both reduced hemoglobin levels and suboptimal immune recovery, providing potential explanations for the observed association (11). These findings further confirm that the first year after ART initiation represents a critical window for immune recovery, highlighting the importance of early identification and intensified follow-up strategies for high-risk individuals (8, 12).

Furthermore, patients with BMI ≥ 24 kg/m^2^ exhibited a significantly higher cumulative probability of immune reconstitution, and BMI remained positively associated with immune recovery after adjustment for other covariates. Although the magnitude of effect was moderate, this finding suggests that baseline nutritional and metabolic status may influence the capacity for long-term immune restoration. Previous studies have shown that undernutrition is associated with chronic inflammation, impaired lymphocyte proliferation, and reduced immune competence in PLWH. In contrast, individuals with adequate nutritional reserves may possess greater physiological capacity to support immune reconstitution following viral suppression (13, 14). Nevertheless, BMI should be interpreted cautiously, as it is a surrogate marker of nutritional and metabolic status and does not capture other important aspects of nutrition, such as dietary intake, micronutrient deficiencies, or socioeconomic determinants, its independent contribution to risk prediction requires further validation in diverse populations.

Importantly, both BMI and hemoglobin are inexpensive, routinely measured, and readily obtainable clinical indicators. Compared with specialized immunological or molecular biomarkers, these parameters are particularly suitable for use in resource-limited settings and large-scale public health programs (15). Their predictive value highlights the importance of considering host nutritional and hematological status in addition to traditional virological and immunological factors. Patients with low BMI and low hemoglobin levels may represent a subgroup requiring closer monitoring after ART initiation. Whether nutritional intervention or anemia management can directly improve immune recovery remains uncertain and should be investigated in future prospective intervention studies.

Regarding prognostic modeling, the present study developed a predictive model based on the independently associated factors identified through multivariable analysis. The model achieved a C-index of 0.713 and an optimism-corrected C-index of 0.711, indicating moderate-to-good discriminative ability and an effective capacity to distinguish patients with successful immune reconstitution from those with immunological non-response. Time-dependent ROC analysis further demonstrated stable predictive performance, with AUC values of 0.849, 0.874, and 0.850 at 1, 2, and 4 years after ART initiation, respectively. These findings suggest that the model possesses robust discriminative performance across short-, intermediate-, and long-term follow-up periods, thereby providing value for both early risk stratification and long-term prognostic assessment (16).

This study has several limitations. First, although we incorporated routinely available demographic, clinical, and laboratory parameters into the predictive model, several potentially important factors were unavailable in our retrospective dataset. These included ART adherence, smoking and alcohol consumption, history of opportunistic infections, inflammatory biomarkers (such as IL-6 and CRP), CD8^+^ T cell activation markers, and socioeconomic characteristics. The absence of these variables may have introduced residual confounding and limited further exploration of the biological mechanisms underlying incomplete immune reconstitution (11).

Second, selection bias should be considered due to the inclusion criteria requiring at least 4 years of ART exposure and sustained virological suppression for ≥3 years. This design excluded individuals with early treatment failure, treatment interruption, death, loss to follow-up, or persistent virological non-suppression before eligibility assessment. Therefore, the study population may represent a relatively stable subgroup of PLWH with better treatment outcomes, which may restrict the generalizability of our findings to patients with shorter ART duration or poorer treatment responses. Nevertheless, this approach was deliberately adopted to focus on long-term immune recovery among virologically suppressed individuals, an increasingly important clinical population in the current ART era (17).

Furthermore, the proportional hazards assumption test indicated that the baseline CD4^+^ T cell count stratification and the WHO clinical stage had time-dependent effects, suggesting that the influence of these factors changed during the follow-up period. This finding is biologically reasonable and is consistent with previous research results, which show that the baseline immune status and disease severity have the greatest impact in the early stage of ART, when CD4^+^ T cell usually recover rapidly, and their influence gradually weakens over time (17, 18).

The early significant inter-group differences observed in the Kaplan–Meier curves further support this explanation. Therefore, the corresponding hazard ratio should be interpreted as the average effect over the entire follow-up period, rather than a fixed time-independent association. Importantly, using time-dependent ROC analysis enables the assessment of predictive performance in different follow-up periods in the presence of potential time-dependent influences. Future studies using time-varying coefficient models or milestone analyses may further clarify the dynamic impact of the baseline immune status on long-term immune recovery.

In summary, this study characterized the dynamic trajectory of immune reconstitution among virologically suppressed HIV-infected individuals, identified several independent determinants of immune recovery, and established a stable predictive model based on routinely available clinical indicators. The model demonstrated satisfactory discrimination and calibration performance and may facilitate early identification of individuals at high risk of immunological non-response, thereby supporting individualized clinical management. External validation in independent cohorts is required before clinical implementation. Future studies integrating multi-omics data and prospective cohort validation are warranted to further optimize the predictive model and elucidate the underlying biological mechanisms of impaired immune reconstitution (19, 20).

## Data Availability

The raw data supporting the conclusions of this article will be made available by the authors, without undue reservation.
